# The SGLT-2 Inhibitors in Personalized Therapy of Diabetes Mellitus Patients

**DOI:** 10.3390/jpm11121249

**Published:** 2021-11-25

**Authors:** Mariana Cornelia Tilinca, Robert Aurelian Tiuca, Ioan Tilea, Andreea Varga

**Affiliations:** “G. E. Palade” University of Medicine, Pharmacy, Science and Technology of Targu Mures, 540142 Targu Mures, Romania; mariana.tilinca@umfst.ro (M.C.T.); ioan.tilea@umfst.ro (I.T.); andreea.varga@umfst.ro (A.V.)

**Keywords:** diabetes mellitus, SGLT-2 inhibitors, antidiabetic agents, cardiovascular outcomes, personalized therapy

## Abstract

Diabetes mellitus (DM) represents a major public health problem, with yearly increasing prevalence. DM is considered a progressive vascular disease that develops macro and microvascular complications, with a great impact on the quality of life of diabetic patients. Over time, DM has become one of the most studied diseases; indeed, finding new pharmacological ways to control it is the main purpose of the research involved in this issue. Sodium–glucose cotransporter 2 inhibitors (SGLT-2i) are a modern drug class of glucose-lowering agents, whose use in DM patients has increased in the past few years. Besides the positive outcomes regarding glycemic control and cardiovascular protection in DM patients, SGLT-2i have also been associated with metabolic benefits, blood pressure reduction, and improved kidney function. The recent perception and understanding of SGLT-2i pathophysiological pathways place this class of drugs towards a particularized patient-centered approach, moving away from the well-known glycemic control strategy. SGLT-2i have been shown not only to reduce death from cardiovascular causes, but also to reduce the risk of stroke and heart failure hospitalization. This article aims to review and highlight the existing literature on the effects of SGLT-2i, emphasizing their role as oral antihyperglycemic agents in type 2 DM, with important cardiovascular and metabolic benefits.

## 1. Introduction

Diabetes mellitus (DM) is a major public health problem, with yearly increasing prevalence. Worldwide, more than 460 million people have DM, with estimations stating that there will be over 700 million cases in the next 20 years [1]. DM is an important risk factor for cardiovascular disease (CVD). Once present, CVD is the main cause of morbidity and mortality in diabetic patients [2,3]. Hence, when managing DM, physicians should be paying attention not only to the proper control of blood glucose, but also to the obtainment of cardiovascular protection. Several glucose-lowering agents, such as metformin or glucagon-like peptide 1 receptor agonists (GLP-1 RAs), have proven important cardiovascular benefits [4,5,6,7,8].

Sodium–glucose cotransporter 2 inhibitors (SGLT-2i) are a modern drug class of glucose-lowering agents, whose use in DM patients has increased in recent years. Currently, the American Diabetes Association (ADA) recommends SGLT-2i as a second-line drug option, after metformin, for managing type 2 DM patients with established CVD [9]. Besides the positive outcomes regarding glycemic control and cardiovascular protection in DM, SGLT-2i have also been associated with metabolic benefits (such as weight loss), blood pressure reduction, and improved kidney function [10,11,12,13]. The positive effects of SGLT-2i have been demonstrated in individuals with and without type 2 DM [14,15].

This article aims to review and highlight the existing literature on the effects of SGLT-2i, emphasizing their role as oral antihyperglycemic agents in type 2 DM, with important cardiovascular and metabolic benefits.

## 2. Mechanism of Action of SGLT-2i and Clinical Effects—A Brief Overview

### 2.1. Mechanism of Action of SGLT-2i

In healthy persons, almost all filtered glucose is reabsorbed (≈180 g daily) and almost no glucose is found in the urine as a consequence of SGLT-2 and SGLT-1 action benefits. SGLT-2 proteins are mainly found in the proximal renal convoluted tubule segment 1, and are responsible for up to 90% of filtered glucose reabsorption, while, in humans, SGLT-1 acts on segment 3 of the proximal tubule and in the intestine, where it reabsorbs the remaining 10% of glucose [16,17,18,19]. The Na^+^/K^+^ ATP pump located on the membrane of the tubular cells provides the energy for glucose reabsorption, which is an active transport mechanism [18]. SGLT-2 can also be found in the brain, liver, thyroid, heart, and skeletal muscle, although in much lower amounts [20]. The physiological renal threshold for glucose reabsorption corresponds to a blood glucose concentration of 180 mg/dL. Patients with DM type 1 and 2 have an increased renal threshold, and, consequently, the expression of SGLT-2 can be up-regulated in diabetic patients, worsening preexisting hyperglycemia [21]. The mechanism of action of SGLT-2i consists of blocking glucose reabsorption at the proximal renal tubule, resulting in glucosuria, osmotic diuresis, and natriuresis, thus reducing blood glucose without stimulating insulin release [22,23]. Currently, in the United States and Europe, there are five SGLT-2i widely used in type 1 and 2 DM patients, which are as follows: canagliflozin (INVOKANA), dapagliflozin (FORXIGA, FARXIGA), empagliflozin (JARDIANCE), ertugliflozin (STEGLATRO), and sotagliflozin (ZYNQUISTA) (Table 1) [18,21,24].

### 2.2. Clinical Effects of SGLT-2i

By suppressing glucose reabsorption and increasing glucosuria (≈75 g glucose/day, which corresponds to almost 300 kcal/day), as well as promoting osmotic diuresis (≈400 mL/day), SGLT-2i reduce blood glucose and promote weight loss [18]. The reduction in fasting and postprandial glucose caused by SGLT-2i is associated with decreased insulin secretion and increased plasma glucagon concentration [25]. Furthermore, by reducing glucotoxicity, SGLT-2i may improve beta-cell function [26,27].

**Figure 1:**
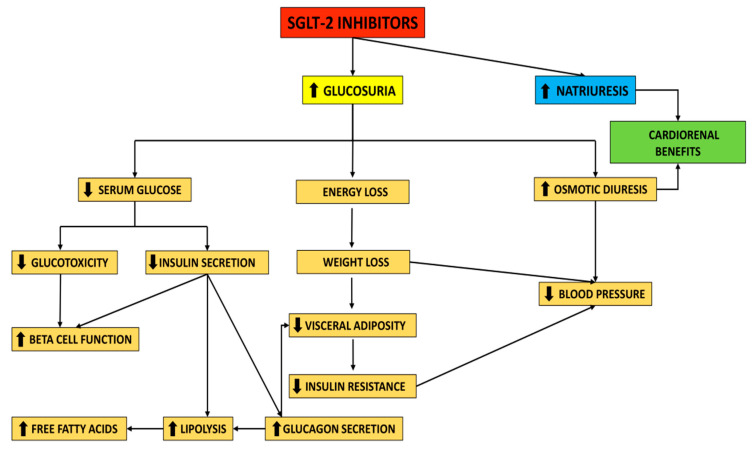
Mechanism of action of SGLT-2 inhibitors.

Taking into consideration the fact that the glucose-lowering effect depends on kidney function, dysfunction of the renal system may alter this effect. Therefore, regarding the glucose-lowering efficacy, therapy with SGLT-2i is not recommended in patients with an estimated glomerular filtration rate (eGFR) <45 mL/min. On the other hand, given that SGLT-2i are associated with positive renal outcomes, therapy with this drug class may be considered in diabetic patients with renal impairment, to improve kidney function [28,29,30]. Decreased insulin secretion results in lipolysis and increased circulating free fatty acids [18]. Glucagon elevation also contributes to lipolysis, and reduces visceral fat [31]. SGLT-2i can reduce the levels of uric acid in a dose-dependent manner [32]. A summary of the mechanism of action and the clinical effects associated with SGLT-2i is illustrated in Figure 1.

## 3. SGLT-2i as Antidiabetic Agents

Diabetes mellitus is a complex metabolic disorder frequently associated with excess weight, hypertension, dyslipidemia, and non-alcoholic fatty liver disease (NAFLD) [33,34]. Moreover, diabetic patients have an increased risk of cardiovascular and renal complications [3,35]. Therefore, an optimal antidiabetic drug should not only possess a good glucose-lowering capacity, but should also exhibit benefits on body weight, blood pressure, lipid profile, NAFLD, cardiovascular function, and renal function. SGLT-2i have been shown to meet many of these criteria. Besides SGLT-2i, glucagon-like peptide 1 (GLP-1) receptor agonists, another modern drug class, exhibit similar benefits in diabetic patients [36,37].

### 3.1. Improving Glycemic Control

SGLT-2i have consistently proven their efficacy in improving glycemic control in patients with type 2 DM in numerous studies, reducing the levels of glycated hemoglobin (HbA1c), and improving the fasting and postprandial glycemic values [38].

Bailey et al. assessed the efficacy and safety of dapagliflozin in patients with type 2 DM poorly controlled with metformin, in a phase 3, double-blind, placebo-controlled trial. A total of 546 patients were randomized to dapagliflozin 2.5, 5, or 10 mg, or a placebo, once daily. It was noted that treatment with dapagliflozin produced greater HbA1c reduction vs. the placebo (−0.67% with dapagliflozin 2.5 mg, −0.70% with dapagliflozin 5 mg, and −0.84% with dapagliflozin 10 mg vs. −0.30% with the placebo; P for all three doses of dapagliflozin vs. placebo: <0.0001) [39]. A meta-analysis of 45 clinical trials, published in 2013 by Vasilakou et al., showed that HbA1c was reduced by 0.79% (95% confidence interval (CI): −0.96% to −0.62%) with SGLT-2i in monotherapy, and by 0.61% (95% CI: −0.69% to −0.53%) with SGLT-2 as an add-on therapy to other antidiabetic agents [40].

In a 26-week, randomized, double-blind, placebo-controlled study, canagliflozin was assessed regarding its safety and efficacy in subjects with type 2 DM and poor glycemic control, uncontrolled with diet and exercise. Canagliflozin (100 and 300 mg) significantly reduced HbA1c compared to the placebo (−0.77% vs. −1.03% vs. −0.14%, respectively; *p* < 0.001 for both doses of canagliflozin) [41]. In a 24-week, randomized, controlled trial, the efficacy and safety of empagliflozin were evaluated when added to linagliptin and metformin in patients with inadequately controlled type 2 DM [42]. Empagliflozin (10 and 25 mg) significantly reduced HbA1c by −0.79% and −0.70%, respectively, versus the placebo. Fasting plasma glucose (FPG) was also significantly reduced by empagliflozin vs. placebo (*p* < 0.001) [40]. A phase 3 study evaluated the efficacy and safety of ertugliflozin plus sitagliptin in patients with type 2 DM inadequately controlled by diet and exercise [43]. After 26 weeks, it was noted that HbA1c was reduced by 1.7%, 1.6%, and 0.4% with ertugliflozin 15 mg plus sitagliptin 100 mg, ertugliflozin 5 mg plus sitagliptin 100 mg, and the placebo, respectively. FPG and postprandial glucose were also significantly reduced in both the ertugliflozin plus sitagliptin groups compared to the placebo group [43].

Shyangdan et al. published a meta-analysis in 2016, which had the objective to indirectly compare SGLT-2i in diabetes treatment. Few differences between SGLT-2i were noted. In monotherapy, canagliflozin 300 mg achieved a greater HbA1c reduction vs. canagliflozin 100 mg (risk ratio (RR): 0.72%) and dapagliflozin 10 mg (RR: 0.63%), without significant differences when compared to empagliflozin [44]. Goring et al. conducted a meta-analysis that assessed the change in HbA1c when adding thiazolidinediones (TZD), sulphonylureas (SU), dipeptidyl peptidase inhibitors (DDP4-i), or dapagliflozin to the treatment of diabetic patients who were uncontrolled with metformin in monotherapy. Dapagliflozin had a treatment effect on HbA1c of −0.08%, relative to DDP4-i, and −0.02%, relative to TZD, with similar HbA1c reduction when compared with SU [45]. Canagliflozin 100 and 300 mg reduced HbA1c by 0.59% and 0.75%, respectively, in a meta-analysis of six randomized controlled trials, which assessed the efficacy and tolerability of canagliflozin as an add-on therapy to metformin in patients with type 2 DM. Moreover, FPG was reduced by 1.49 mmol/L (canagliflozin 100 mg) and 1.80 mmol/L (canagliflozin 300 mg) [46].

According to a meta-analysis of randomized controlled trials, published in 2014, when added to other antidiabetic drugs, dapagliflozin reduced HbA1c and FPG by 0.52% and 20 mg/dL, respectively [47]. A meta-analysis that included 38 trials and 23,997 participants showed that SGLT-2i improved glucose control, achieving a decrease in HbA1c between 0.6–0.9% and a decrease in FPG between 1.1–1.9 mmol/L [48]. The efficacy of ertugliflozin was assessed when given to patients with type 2 DM, who were treated with metformin [49]. After 26 weeks, ertugliflozin 5 and 15 mg reduced HbA1c by 0.7% and 0.9%, respectively [49]. A randomized, double-blind, placebo-controlled 4-week study compared dapagliflozin 10 mg/day with a placebo in adult patients with type 2 DM, monitoring a 24 h glycemic profile using continuous glucose monitoring. After 4 weeks, dapagliflozin 10 mg/day achieved a mean glucose reduction of 18.2 mg/dL vs. 5.8 mg/dL increase with a placebo (*p* < 0.001) [50].

In a 24-week, randomized, controlled trial, empagliflozin 10 or 25 mg was added to pioglitazone in patients with type 2 DM, with or without metformin. Empagliflozin achieved a HbA1c reduction of −0.6% (10 mg) and −0.7% (25 mg) vs. −0.1% with a placebo (P for both doses of empagliflozin < 0.001) [51]. FPG was reduced by −0.94 mmol/L (10 mg) and −1.22 mmol/L (25 mg) vs. +0.36 mmol/L with a placebo (P for both doses of empagliflozin < 0.001) [51]. Empagliflozin 10 and 25 mg was evaluated regarding its efficacy when added to liraglutide 0.9 mg/day in a 52-week, randomized, phase 4 trial [52]. After 52 weeks, empagliflozin 10 and 25 mg improved HbA1c by 0.55% and 0.77%, respectively. FPG was also reduced by 32.5 mg/dL and 36 mg/dL, respectively [52]. Terra et al. investigated the efficacy of ertugliflozin 5 and 15 mg in patients with diabetes that was were inadequately controlled with diet and exercise [53]. After 26 weeks, the treatment with ertugliflozin significantly reduced HbA1c from the baseline by 0.99% (5 mg) and 1.16% (15 mg), improving FPG and postprandial glucose as well [53].

A summary of the presented studies, regarding the effects of SGLT-2i on improving glycemic control, is illustrated in Supplementary Table S1.

### 3.2. Body Weight Reduction Benefits and Effects on Blood Pressure

SGLT-2i also have weight reduction benefits in diabetic patients, which may help in diminishing insulin resistance, reducing complications, and improving quality of life. Body weight loss was observed in patients with type 2 DM who were taking SGLT-2i as a monotherapy or in combination with other antidiabetic agents.

Regarding body composition, Bolinder et al. showed, through dual-energy X-ray absorptiometry, that dapagliflozin reduced total body weight by mostly reducing fat mass [54]. Moreover, dapagliflozin reduced visceral and subcutaneous adiposity, according to a magnetic resonance imaging sub-study [54]. On the other hand, Fadini et al. found that, compared to a placebo, dapagliflozin did not affect fat mass when the body composition was analyzed by bio-impedenzometry [55].

Ferrannini et al. observed that, even though glucosuria is persistent over time, body weight loss reaches a plateau, given that chronic glucosuria may induce an adaptive increase in energy intake. Therefore, SGLT-2i may induce greater weight loss if combined with reduced caloric intake [56]. Ji et al. noted a dose-dependent weight loss effect for dapagliflozin 5 mg and 10 mg compared with a placebo (−1.64 kg vs. −2.25 kg vs. −0.27 kg, respectively) [57]. Kaku et al. reported similar results, regarding body weight reduction effects, for dapagliflozin 5 mg and 10 mg compared with a placebo (−2.13 kg vs. −2.22 kg vs. −0.84 kg, respectively) [58]. Combining SGLT-2i with other agents that possess weight reduction benefits via different mechanisms may lead to major weight loss [11]. The co-administration of dapagliflozin once daily (10 mg) and exenatide, a member of the GLP-1 RAs family, once weekly (2 mg) resulted in a greater mean bodyweight loss in patients with type 2 DM than that achieved with monotherapies alone. The change in baseline weight was −3.41 kg for the exenatide plus dapagliflozin group, −1.54 kg for the exenatide group, and −2.19 kg for the dapagliflozin group [59]. Strojek et al. observed that adding dapagliflozin to glimepiride, a member of the sulphonylureas family, improved body weight compared with a placebo. Dapagliflozin 2.5, 5, and 10 mg/day added to glimepiride 4 mg/day produced a weight loss of −1.36, −1.54, and −2.41 kg, respectively, vs. −0.77 kg with a placebo [60].

Canagliflozin exhibits positive and dose-dependent effects on body weight, HbA1c, and systolic blood pressure reduction. Patients who achieved greater weight loss also achieved greater reductions in HbA1c and systolic blood pressure. A weight loss of 1% was associated with a 0.045% reduction in HbA1c and a 0.62 mmHg reduction in systolic blood pressure [61]. In the same direction, in a recent meta-analysis, SGLT-2i was were proven to have a mean reduction, both in systolic and diastolic blood pressure, assessed by 24 h ambulatory blood pressure monitoring. Independent of the SGLT-2i dose, this effect is comparable with a low dose of hydrochlorothiazide [62]. According to the results of a the Systolic Blood Pressure Intervention trial (SPRINT trial), in hypertensive patients without diabetes, who have increased cardiovascular risk, targeting a systolic blood pressure of ≤120 mmHg (intensive strategy), rather than ≤140 mmHg (standard strategy), was associated with lower incidence of major adverse cardiovascular events and all-cause mortality [63,64]. Buckley et al. conducted a study to determine the effects of intensive blood pressure on cardiovascular outcomes in subjects with type 2 DM and additional risk factors for cardiovascular disease. Intensive blood pressure treatment reduced the incidence of cardiovascular death, nonfatal myocardial infarction, revascularization, and heart failure (HF) [65]. In a review conducted by Oliva et al., SGLT-2i (dapagliflozin and canagliflozin) were associated with a reduction of 4–10 mmHg in systolic blood pressure in both hypertensive and normotensive patients with type 2 DM [66]. Tikkanen et al. demonstrated that empagliflozin produced a significant reduction in 24 h systolic and diastolic blood pressure in patients with type 2 DM [67]. Moreover, empagliflozin also produced a significant reduction in 24 h systolic (−5.1 mmHg) and diastolic (−2.0 mmHg) blood pressure in non-diabetic patients, without acute changes regarding renal oxygenation [68]. Therefore, although this modern drug class is not approved as an antihypertensive agent, by reducing body weight and high blood pressure, SGLT-2i have a positive impact on reducing both cardiovascular and renal risk, regardless of the presence or absence of diabetes.

### 3.3. SGLT-2i and Cardiorenal Continuum

**Figure 2:**
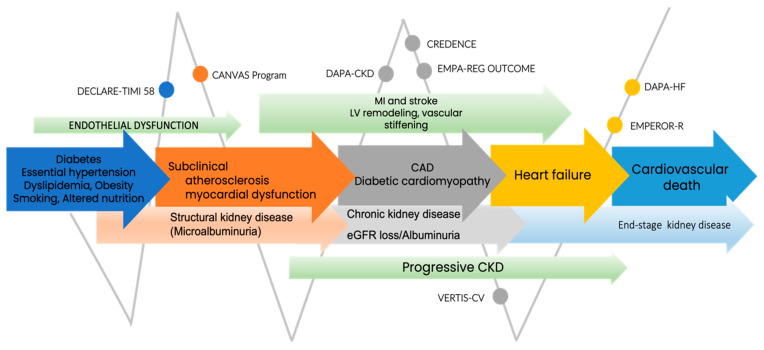
SGLT-2 randomized studies in cardiorenal continuum. Abbreviations: CAD, coronary artery disease; CKD, chronic kidney disease; eGFR, estimated glomerular filtration rate; LV, left ventricle; MI, myocardial infarction.

Diabetic patients are prone to developing atherosclerotic cardiovascular disease (ASCVD), HF, and renal disease. The pathophysiological chain of the cardiorenal continuum illustrates a continuous progression up to the end stage of heart disease; with a focus on a diabetic subgroup, such as type 2 DM, this is the cardinal point in the cardiorenal continuum, as is shown in Figure 2 [69]. Cardioprotective outcomes in type 2 DM patients mediated by SGLT-2i are related to systemic and direct myocardial effects. In cardiac homeostasis, the main effects address decreasing myocardial fibrosis/steatosis, reducing cardiac and vascular inflammation, and improving systolic and diastolic myocardial function [70]. SGLT-2i have moderate benefits in patients with baseline ASCVD, decreasing the major adverse cardiovascular events (MACE) by 14% [14].

Referring to the effects of empagliflozin in type 2 DM patients with coronary artery disease, Bilgin et al. stated, in the SUPER GATE study, that the use of this SGLT-2i significantly reduced the anthropometric indices (body weight and mass index, and waist and hip circumferences) associated with type 2 DM. In this subset of patients, the metabolic parameters, including low-density lipoprotein cholesterol levels, were improved, along with targeted blood pressure values and heart rate. The kidney function, assessed by serum creatinine levels and eGFR, was not significantly modified [71].

The multicenter, double-blind, randomized controlled VERTIS-CV trial tested the effect of ertugliflozin on patients with type 2 DM and established ASCVD, and showed the non-inferiority of this SGLT-2i compared to a placebo, in terms of MACE and reduction in risk of the first hospitalization for HF, but not for known HF patients [72]. The cardioprotection mechanisms of SGLT-2i use were conclusively proved in in vivo models by dapagliflozin, which decreases myocardial fibrosis through the inhibition of fibroblast multiplication in the endothelial-to-mesenchymal transition (EndMT) via AMPKα-mediated inhibition of TGF-β/Smad signaling [73]. In ischemic myocardial injury in nondiabetic HF, with reduced ejection fraction (HFrEF) porcine models, empagliflozin showed improved diastolic dysfunction, alleviating left ventricle and cardiomyocyte stiffness [74].

Chronic kidney disease (CKD) is recognized in 30% of patients with type 1 DM and 40% of patients with type 2 DM [75]. Initially, empagliflozin, canagliflozin, and dapagliflozin demonstrated nephroprotection in DM patients without advanced CKD [76,77,78]. Clinical trials and real-world clinical practice data of SGLT-2i use in patients with type 2 DM, evaluating renal outcome, showed both a reduction in the loss of eGFR and end-stage kidney disease [79]. In patients with CKD in different stages, independent of diabetes condition, DAPA-CKD (Dapagliflozin and Prevention of Adverse Outcomes in Chronic Kidney Disease) and CREDENCE (Canagliflozin and Renal Events in Diabetes with Established Nephropathy Clinical Evaluation) trials walk one through renal protection. The CREDENCE trial confirmed the use of canagliflozin down to an eGFR of 30 mL/min/1.73 m^2^. Nonetheless, it remains to be clarified whether this beneficial effect can be attributed to the drug itself, or whether it is an SGLT-2 class effect [80,81,82]. Ertugliflozin reduced renal risk by 19%, but still did not prove significant benefits in composite renal outcomes, including renal death, need for dialysis or kidney transplantation, or a two-fold increase in serum creatinine, in the VERTIS CV study [72].

#### 3.3.1. The Era of SGLT-2i Benefits in Heart Failure

The risk of developing HF in DM patients is at least two times higher compared to non-diabetic patients [83]. The potential mechanism by which SGLT-2i intervene in both the preserved and reduced HF risk of type 2 DM patients is related to multiple pathophysiological processes addressing three major directions, which are as follows:

1. Decreased preload (increased osmotic diuresis and natriuresis);

2. Decreased afterload (lowered blood pressure, reduced arterial stiffness, and vascular resistance);

3. Strengthening of myocardial contractility (inhibition of cardiomyocyte Na^+^/H exchanger; increased myocardial energetics, systolic and diastolic function, cardiac output, heart rate, O^2^ consumption, and mediated coronary blood flow; reduction in myocardial Ca^2+^/calmodulin-dependent protein kinase II activity and left ventricular mass) [70,84].

SGLT-2i demonstrated positive effects on the scaling down of admission rates in HF patients, and the lowering of CV (cardiovascular) and all-cause mortality in diabetic patients [72,78,85,86,87]. The use of three SGLT-2i (empagliflozin, canagliflozin, and dapagliflozin) have a Class 1 level of evidence A, in that they lower the risk of HF hospitalization in DM patients by 35%, 33%, and 27%, respectively, as stated in the 2019 European Society of Cardiology Guidelines on diabetes, pre-diabetes, and cardiovascular diseases [88].

The first clinical trial exploring the effectiveness of SGTL-2i on cardiovascular outcomes in HFrEF patients, EMPA-REG OUTCOME (Empagliflozin Cardiovascular Outcome Event Trial in Type 2 Diabetes Mellitus Patients), met the primary outcome in lowering the deaths from cardiovascular causes, nonfatal myocardial infarction or stroke, and the secondary outcome for hospitalization for HF (hHF) [70]. The 10 mg, once daily dose efficacy of empagliflozin, as an add-on to the optimal HF therapy, was explored in patients with HFrEF, New York Heart Association (NYHA) functional class II–IV, in the EMPEROR Reduced trial [89]. Empagliflozin reduced the risk of death due to CV events and acute HF episodes by 5.3% compared to a placebo, *p* < 0.001, at a 16-month follow-up. No significant differences were observed between empagliflozin-treated patients and patients treated with a placebo, in terms of hypoglycemic episodes, amputation of the lower limb, and bone fractures [89]. Empagliflozin effects in patients with HF with preserved ejection fraction (HFpEF), with or without type 2 DM, were investigated in EMPEROR—Preserved (Empagliflozin Outcome Trial in Patients with Chronic Heart Failure with Preserved Ejection Fraction). The novelty of the results consists of demonstrating the reduction in the combined risk of cardiovascular death or hHF [90].

The CANVAS study program (Canagliflozin Cardiovascular Assessment Study and Canagliflozin Cardiovascular Assessment Study—Renal) also confirmed the reduction in hHF in patients with type 2 DM and ASCVD, or patients at high risk for CV occurrence, and also confirmed the reduction in hospitalization of patients with no past events of HF. Canagliflozin diminished the risk of death from cardiovascular causes, nonfatal myocardial infarction, or stroke; however, an increased risk of amputation was recorded compared to a placebo [82].

For dapagliflozin, the DAPA-HF (Dapagliflozin and Prevention of Adverse Outcomes in Heart Failure) study showed a reduction in hospitalization for HF and death from cardiovascular causes compared to a placebo, though this favored patients with NYHA II functional class compared to those with NYHA III or IV functional class [91]. Positive results and a beneficial protection profile of dapagliflozin were also observed in non-diabetic patients with HF and CKD [92].

A summary of the presented clinical trials in Section 3.3 and Section 3.3.1 is illustrated in Supplementary Table S2.

The results of the following ongoing trials will offer evidence-based data on the role of dapagliflozin in selected outcomes: Dapagliflozin Evaluation to Improve the LIVEs of Patients With Preserved Ejection Fraction Heart Failure (DELIVER), DAPA ACT HF-TIMI 68, Efficacy and Safety of Dapagliflozin in Acute Heart Failure (DICTATE-AHF), Dapagliflozin Heart Failure Readmission Study, DAPA MI Study, and Effectiveness of Dapagliflozin for Weight Loss [93,94,95,96,97,98].

## 4. Conclusions

The recent understanding of the pathophysiological pathways of SGLT-2i places this class of drugs towards a particularized, patient-centered approach, moving away from the well-known glycemic control strategy. SGLT-2i exhibit beneficial outcomes regarding glycemic control in type 2 DM, while also exhibiting weight loss benefits; the latter effect is especially observed when combined with other weight-reduction agents. SGLT-2i, by promoting weight loss and positively impacting high blood pressure, may have an additional cardiorenal protective role. Moreover, SGLT-2i have been shown not only to reduce death from cardiovascular causes, but also to reduce the risk of stroke and hospitalization due to heart failure. As a result of the positive cardiovascular and renal outcomes reported by numerous studies, SGLT-2i might be considered as one of the most reliable and efficient treatment options in diabetic patients nowadays, given that one of the main goals in treating type 2 DM is to preservee and improve cardiorenal function. Relying on the results of SGLT-2i studies, with beneficial effects on cardiovascular outcomes and mortality in patients with type 2 DM, as well as in non-diabetic patients, prescribing this class of drugs in medical practice is becoming a priority in personalized medicine.

## Figures and Tables

**Table 1:** List of the currently most used SGLT-2i in the United States and Europe.

Name	Available Doses (Milligrams)	Route of Administration
Canagliflozin (INVOKANA^®^)	100, 300	Oral, q.a.m
Dapagliflozin (FORXIGA^TM^, FARXIGA^TM^)	5, 10	Oral, q.a.m
Empagliflozin (JARDIANCE^®^)	10, 25	Oral, q.a.m
Ertugliflozin (STEGLATRO^®^)	5, 15	Oral, q.a.m
Sotagliflozin (ZYNQUISTA™)	200	Oral, q.a.m
Abbreviations	q.a.m, every morning	

## Data Availability

Not applicable.

## Supplementary Materials

The following are available online at https://www.mdpi.com/article/10.3390/jpm11121249/s1, Table S1: summary of SGLT-2i effects on glycemic control as found in several studies. Table S2: summary of SGLT-2i cardiorenal outcomes as reported in randomized clinical trials.
