# Supplementary material for: The SGLT-2 Inhibitors in Personalized Therapy of Diabetes Mellitus Patients
Source: J Pers Med. 2021 Nov 25;11(12):1249. doi: 10.3390/jpm11121249 (PMC8708213; doi:10.3390/jpm11121249)
Supplement: Supplementary file 1 [file jpm-11-01249-s001.zip › table S1.pdf]

Table S1. Summary of SGLT-2i effects on glycemic control as found in several studies

First author	Aim	Main results
Bailey[39]	To assess the efficacy and safety of dapagliflozin in subjects with poorly controlled type 2 DM under metformin	Dapagliflozin 2.5 mg, 5 mg, and 10 mg produced a significant greater HbA1c reduction vs placebo (-0.67% vs -0.70% vs -0.84% vs -0.30%)
Vasilakou[40]	To assess the efficacy and safety of SGLT-2i in adults with type 2 DM	SGLT-2i in monotherapy reduced Hb1c by 0.79% and by 0.61% as add-on therapy to other antidiabetic agents
Stenlöf[41]	To assess the efficacy and safety of canagliflozin in type 2 DM subjects with poor glycemic control under diet and exercise	Canagliflozin 100 and 300 mg produced a significant greater HbA1c reduction vs placebo (-0.77% vs -1.03% vs -0.14%)
Søfteland[42]	To assess the efficacy and safety of empagliflozin when added to linagliptin and metformin in subjects with poorly controlled type 2 DM	Empagliflozin 10 mg and 25 mg significantly reduced HbA1c vs placebo<br><br>Empagliflozin significantly reduced FPG vs placebo
Miller[43]	To assess the efficacy and safety of ertugliflozin plus sitagliptin in subjects with poorly controlled type 2 DM under diet and exercise	Ertugliflozin 15 mg + Sitagliptin 100 mg reduced HbA1c by 1.7%<br><br>Ertugliflozin 5 mg + Sitagliptin 100 mg reduced HbA1c by 1.6%<br><br>Ertugliflozin significantly reduced FPG and postprandial glucose
Shyangdan[44]	To indirectly compare SGLT-2i in the treatment of type 2 DM	Canagliflozin 300 mg achieved a greater HbA1c reduction vs canagliflozin 100 mg and vs dapagliflozin<br><br>Canagliflozin 300 mg produced no significant differences when compared to empagliflozin
Goring[45]	To assess HbA1c change when TZD, SU, DDP4-i, or dapagliflozin were added to metformin in subjects with poorly controlled type 2 DM	The treatment effects of dapagliflozin on HbA1c was -0.08% relative to DDP4-I, -0.02% relative to TZD and 0.00% relative to SU
Yang[46]	To assess the efficacy and tolerability of canagliflozin as add-on therapy to metformin in subjects with type 2 DM	Canagliflozin 100 and 300 mg reduced HbA1c by 0.59% and 0.75%, respectively<br><br>Canagliflozin 100 and 300 mg reduced FPG by 1.49 mmol/L and 1.80 mmol/L, respectively
Sun[47]	To assess if dapagliflozin is synergistic with other antidiabetic drugs without body weight gain	Dapagliflozin reduced HbA1c by 0.52% and FPG by 20 mg/dL when added to other antidiabetic drugs
Zaccardi[48]	To assess the efficacy and safety of SGLT-2i in adults with type 2 DM	SGLT-2i decreased HbA1c by 0.6-0.9% and FPG by 1.1-1.9 mmol/L
Rosenstock[49]	To assess the efficacy and safety of ertugliflozin in subjects with poorly controlled type 2 DM under metformin	Ertugliflozin 5 and 15 mg reduced HbA1c by 0.7% and 0.9%, respectively
Henry[50]	To compare dapagliflozin 10 mg/day vs placebo by monitoring 24-hour glycemic profile using continuous glucose monitoring in adult subjects with type 2 DM	Dapagliflozin 10 mg/day achieved a mean glucose reduction by 18.2 mg/dL while placebo had an increase by 5.8 mg/dL

Kovacs[51]	To assess the efficacy and safety of empagliflozin as add-on to pioglitazone and/or metformin in subjects with type 2 DM	Empagliflozin 10 mg/day and 25 mg/day achieved a HbA1c reduction of -0.6% and -0.7%, respectively vs -0.1% with placebo<br><br>Empagliflozin 10 mg/day and 25 mg/day achieved a FPG reduction of -0.94 mmol/L and -1.22 mmol/L, respectively vs +0.36 mmol/L with placebo
Terauchi[52]	To assess the safety, tolerability, and efficacy of empagliflozin as add-on to liraglutide in subjects with type 2 DM	Empagliflozin 10 mg/day and 25 mg/day reduced HbA1c by -0.55% and -0.77%, respectively<br><br>Empagliflozin 10 mg/day and 25 mg/day reduced FPG by -32.5 mg/dL and -36 mg/dL, respectively
Terra[53]	To assess the safety and efficacy of ertugliflozin as monotherapy in subjects with type 2 DM	Ertugliflozin 5 mg/day and 15 mg/day reduced HbA1c by -0.99% and by -1.16%, respectively<br><br>Ertugliflozin 5 mg/day and 15 mg/day reduced FPG by -1.92 mmol/L and by -2.44 mmol/L, respectively
Abbreviations	DDP4-i, dipeptidyl peptidase inhibitors; FPG, fasting plasma glucose; HbA1c, glycated hemoglobin; mg, milligram; SGLT-2i, sodium-glucose cotransporter-2 inhibitors; SU, sulphonylureas; TZD, thiazolidinediones; type 2 DM, type 2 diabetes mellitus; vs, versus	
