# Supplementary material for: The SGLT-2 Inhibitors in Personalized Therapy of Diabetes Mellitus Patients
Source: J Pers Med. 2021 Nov 25;11(12):1249. doi: 10.3390/jpm11121249 (PMC8708213; doi:10.3390/jpm11121249)
Supplement: Supplementary file 1 [file jpm-11-01249-s001.zip › table S2.pdf]

Table S2. Summary of SGLT-2i cardiorenal outcomes as reported in randomized clinical trials

Clinical trial	Design	Aim	Main outcomes
DECLARE-TIMI 58[78]	Multinational, randomized, double-blind, placebo-controlled phase IIIB trial<br><br>Subjects with type 2 DM who had or were at risk for ASCVD received either dapagliflozin (10 mg/day) or placebo	To test if long-term treatment with dapagliflozin reduces one or both of primary endpoints: (1) MACE defined as cardiovascular death, myocardial infarction, or ischemic stroke or (2) the incidence of cardiovascular death or hHF	Number of subjects: 17.160. *Dapagliflozin group – 8582* *Placebo group – 8578*<br><br>Dapagliflozin did not result in a higher or lower rate of MACE (8.8% in dapagliflozin group vs 9.4% in placebo group). However, it did result in a lower rate of cardiovascular death or hHF (4.9% vs 5.8%)
CANVAS program[77,82]	Two double-blind, placebo-controlled, randomized trials, phase III (*CANVAS*) and phase IV (*CANVAS-R*) *CANVAS*: Subjects with type 2 DM with history of cardiovascular events or increased risk for cardiovascular events were randomized to treatment with 1 of 2 doses of canagliflozin (100 or 300 mg) or placebo. *CANVAS-R:* Subjects with type 2 DM with a history of cardiovascular events or increased risk for cardiovascular events were randomized to canagliflozin (100 mg for 13 weeks then might be increased to 300 mg) or placebo	To assess canagliflozin vs placebo in the treatment of subjects with type 2 DM regarding its cardiovascular risk for MACE and the progression of albuminuria	Number of subjects: 10.142 *CANVAS* – 4330 *CANVAS-R* – 5812<br><br>Canagliflozin was associated with reduced risk of sustained loss of kidney function<br><br>Canagliflozin reduced hHF in patients with type 2 DM and ASCVD or at high risk for cardiovascular events<br><br>Canagliflozin reduced the risk of death from MACE<br><br>Increased risk of amputation compared to placebo
DAPA-CKD[80]	Multicentre, randomized, double-blind, placebo-controlled phase III trial	To assess the effect of dapagliflozin on renal outcomes and cardiovascular mortality in subjects with CKD	Number of subjects: 4304 *Dapagliflozin group – 2152* *Placebo group – 2152*<br><br>Dapagliflozin treated subjects had lower risk of sustained decline in eGFR of at least 50%,

	Subjects with eGFR between 25-75 ml/minute/1.73 m^2, with or without type 2 DM, received either dapagliflozin (10 mg/day) or placebo		end-stage kidney disease, or death from renal or cardiovascular causes
CREDENCE[81]	Multicentre, randomized, double-blind, placebo-controlled phase III trial<br><br>Subjects with type 2 DM and albuminuric CKD received either canagliflozin (100 mg/day) or placebo	To assess whether canagliflozin has renal and vascular protective properties in reducing the progression of CKD in subjects with type 2 DM	Number of subjects: 4401<br>*Canagliflozin group – 2202*<br>*Placebo group – 2199*<br><br>Treatment with canagliflozin reduced the risk of kidney failure and cardiovascular events<br><br>Canagliflozin group had a lower risk of MACE and hHF
EMPA-REG OUTCOME[51,76,85,86]	Multicentre, randomized, double-blind, placebo-controlled phase III trial<br><br>Subjects with type 2 DM and ASCVD received either empagliflozin (10 mg/day or 25 mg/day) or placebo	To assess the safety of treatment with empagliflozin in subjects with type 2 DM and high cardiovascular risk	Number of subjects: 7020<br>*Empagliflozin 10 mg group – 2345*<br>*Empagliflozin 25 mg group – 2342*<br>*Placebo group – 2333*<br><br>Empagliflozin reduced the risk of cardiovascular death by 38%, death by any cause by 32%, and hHF by 35%<br><br>Empagliflozin showed consistent benefits in subjects with and without baseline heart failure<br><br>Empagliflozin was associated with reduced progression of CKD compared with placebo
VERTIS-CV[72]	Multicentre, randomized, double-blind, placebo-controlled phase III trial<br><br>Subjects with type 2 DM and ASCVD received either ertugliflozin (5 mg/day or 15 mg/day) or placebo	To assess the cardiovascular safety of ertugliflozin in subjects with type 2 DM and established vascular disease	Number of subjects: 8246<br>*Ertugliflozin group – 5499*<br>*Placebo group – 2747*<br><br>Ertugliflozin was not inferior to placebo regarding MACE (11.9% vs 11.9%)<br><br>Death from cardiovascular causes or hHF occurred in 8.1% of subjects in the ertugliflozin group and in 9.1% of subjects in the placebo group<br><br>Amputation occurred in 2.0% of subjects treated with 5 mg of

			ertugliflozin, in 2.1% of subjects treated with 15 mg of ertugliflozin, and in 1.6% of subjects who received placebo
DAPA-HF[15]	Multicentre, randomized, double-blind, placebo-controlled phase III trial<br><br>Subjects with NYHA class II, III, or IV heart failure and an ejection fraction of ≤ 40% received as add-on either dapagliflozin (10 mg/day) or placebo	To assess the effect of dapagliflozin in subjects with established heart failure and reduced ejection fraction and the incidence of worsening heart failure and/or cardiovascular death, regardless of the presence or absence of type 2 DM	Number of subjects: 4744<br>*Dapagliflozin group – 2373*<br>*Placebo group – 2371*<br><br>Dapagliflozin treated subjects had a lower risk of worsening heart failure (10% vs 13.7%) or death from cardiovascular causes (9.6% vs 11.5%) compared with placebo<br><br>Results in subjects with type 2 DM were similar to those without type 2 DM
EMPEROR-R[89]	Randomized, double-blind, placebo-controlled phase III trial<br><br>Subjects with NYHA class II, III, or IV heart failure and an ejection fraction of ≤ 40% received as add-on either empagliflozin (10 mg/day) or placebo	To assess the safety and efficacy of empagliflozin in subjects with heart failure and reduced ejection fraction	Number of subjects: 3730<br>*Empagliflozin group – 1863*<br>*Placebo group – 1867*<br><br>Adding empagliflozin to chronic therapy for heart failure resulted in lower risk of cardiovascular death or hHF compared with placebo (19.4% vs 24.7%)<br><br>Empagliflozin-treated subjects had a lower risk of serios renal outcomes with a slower annual decline in eGFR compared with placebo-treated subjects (-0.55 vs -2.28 ml/minute/1.73 m^2)
EMPEROR-P[90]	Randomized, double-blind, placebo-controlled phase III trial<br><br>Subjects with NYHA class II, III, or IV heart failure and an ejection fraction of > 40% received as add-on either empagliflozin (10 mg/day) or placebo	To assess if empagliflozin reduces the hHF and improves survival in subjects with chronic heart failure.	Number of subjects: 5988<br>*Empagliflozin group – 2997*<br>*Placebo group – 2991*<br><br>Empagliflozin-treated subjects had a lower incidence of cardiovascular death and hHF compared with placebo (13.8% vs 17.1%)<br><br>The positive outcomes were consistent regardless of the presence or absence of diabetes
Abbreviations	ASCVD, atherosclerotic cardiovascular disease; CKD, chronic kidney disease; eGFR, estimated glomerular filtration rate; hHF, hospitalization for heart failure; MACE, major adverse cardiovascular events; mg, milligram; NYHA, New York Heart Association; SGLT-2i, sodium-glucose cotransporter-2 inhibitors; type 2 DM, type 2 diabetes mellitus; vs, versus		
